# Hematopoietic Stem Cell Transplantation for Systemic Lupus Erythematosus

**DOI:** 10.1155/2012/380391

**Published:** 2012-08-30

**Authors:** Alberto M. Marmont du Haut Champ

**Affiliations:** Division of Hematology and Stem Cell Transplantation, IRCCS Azienda Ospedaliera Universitaria San Martino-IST, Genoa, Italy

## Abstract

Two streams of research are at the origin of the utilization of hematopoietic stem cell transplantation (HSCT) for severe autoimmune diseases (SADs). The allogeneic approach came from experimental studies on lupus mice, besides clinical results in coincidental diseases. The autologous procedure was encouraged by researches on experimental neurological and rheumatic disorders. At present the number of allogeneic HSCT performed for human SADs can be estimated to not over 100 patients, and the results are not greatly encouraging, considering the significant transplant-related mortality (TRM) and the occasional development of a new autoimmune disorder and/or relapses notwithstanding full donor chimerism. Autologous HSCT for refractory SLE has become a major target. Severe cases have been salvaged, TRM is low and diminishing, and prolonged clinical remissions are obtainable. Two types of immune resetting have been established, “re-education” and regulatory T cell (Tregs) normalization. Allogeneic HSCT for SLE seems best indicated for patients with disease complicated by an oncohematologic malignancy. Autologous HSCT is a powerful salvage therapy for otherwise intractable SLE. The duration of remission in uncertain, but a favorable response to previously inactive treatments is a generally constant feature. The comparison with new biological agents, or the combination of both, are to be ascertained.

## 1. Introduction

Hematopoietic stem cell transplantation (HSCT), especially in its autologous form, has become a significant treatment modality for severe autoimmune diseases [1–9] (SADs), and more specifically for systemic lupus erythematosus (SLE) and the antiphospholipid syndrome [10–14]. Most of the evidence concerns the hematopoietic lineage. However, the utilization of another distinct lineage, consisting of mesenchymal stromal cells (MSC), is also becoming a promising sector in the field of regenerative medicine and immune disorders [15, 16]. Bone mesenchymal stem cells (BMSC) are not transplanted along with hematopoiesis in standard marrow and blood transplantation [17]. However there are 2 important studies in which allogeneic MSC were transplanted in patients with severe-refractory SLE. In both, no pretransplant conditioning was utilized because of the well-known low MSC immunogenicity. Fifteen lupus patients received 1 intravenous infusion of 1 × 10^6^ MSC/Kg, and both the clinical (by SLEDAI score) and the laboratory (DNA, ANA) results were clearly favorable [18]. Another study by the same investigators was performed with umbilical MSC, utilizing low-dose cyclophosphamide (CY) conditioning in about half of them, in 16 lupus patients, again with significant amelioration in SLEDAI and laboratory results [19], which were accompanied by an increase in peripheral Treg cells, a feature that was also found in other SLE patients treated with conventional autologous HSCT [20]. However, notwithstanding these recent and encouraging results, the bulk of classical evidence provenes from the two traditional procedures of hematopoietic stem cell transplantation, allogeneic (allo-HSCT) and overwhelmingly autologous (auto-HSCT).

## 2. Historical Perspective and Rationale

Two streams of research, experimental and clinical, are at the origin of the increasing utilization of HSCT, autologous and allogeneic, for SADs. Somewhat unexpectedly, although the initial evidence was in favor of the allogeneic procedure, it was the autologous one that attained greater consensus and much greater utilization. The history of these earlier studies has been described in detail elsewhere [21]. It all started with animal experiments.

In memorable studies it was shown that the transfer of spleen cells or whole bone marrow cells from New Zealand Black (NZB; H-2^d^) mice to antilymphocyte globulin treated BALB-L, H-2^d^ id irradiated mice was capable of reproducing the donors' murine lupus [22, 23]. These original experiments have been considerably enlarged by recent studies by Smith-Berdan et al. [24], who obtained the reversal of murine lupus by nonmyeloablative transplantation of purified allogeneic HSC, a procedure which they advocated also for human SADs. Other studies demonstrated that the B lymphoid precursors from B/W F_1_ bone marrow (BM) cultures reproduced the disease in SCID mice [25]. In a series of consecutive experimental investigations Ikehara came to the conclusion that animal ADs were stem cell diseases [26, 27].

Allogeneic HSCT received a vigorous impulse also from the clinic. There is a series of reports of patients harboring an AD and having developed a hematological malignancy, who were cured of both diseases following an allogeneic HSCT. Such patients go under the definition of coincidental diseases, and a detailed review has been published [28]. These results were encouraging, but in other ones the AD persisted in spite of cure of the malignancy. Thus the initial enthusiasm for the allogeneic procedure has decreased considerably [29].

The apparent paradox of treating patients with ADs with autotransplantation, that is giving them back, with or without T cell depletion, their own HSC originated with the pioneering experimental investigations by van Bekkum and his group, who treated successfully experimental ADs, such as experimental autoimmune encephalitis (EAE: the experimental model of multiple sclerosis, MS) and adjuvant arthritis (AA: the experimental model of rheumatoid arthritis, RA) with first syngeneic and then autologous BM transplants [30]. However, these results were obtained in the induced rather than in the spontaneous animal ADs [26, 27]. These apparently paradoxical but encouraging results considerably strengthened the philosophy of auto-HSCT for human SADs, which has grown almost exponentially in the last 2 decades. Systemic lupus erythematosus (SLE) is widely considered as the paradigm of ADs and autologous HSCT for patients with severe refractory lupus disease was first proposed by myself in 1993 [31]. This proposal was soon after extended to all SADs [32] and updated guidelines have been published recently by Sullivan et al. [8].

## 3. Allogeneic Transplantation

Two important conferences have analyzed the possible indications for allo-HSCT in ADs [33, 34]. In the Position Paper of 2005 [34] the potential results and the attending risks of allo-HSCT for SADs have been discussed in detail. The capability of a 1-time delivery of a curative therapeutic strategy was considered as “appealing.” A comprehensive recent review of clinical results has been published by Gratwohl [35]. However the number of SLE patients having undergone allo-HSCT is minimal and reference must be made to the greater experience in SADs in general.

A retrospective EBMT study [36] identified 35 patients having received 38 allogeneic transplants for various SADs, including 2 cases of SLE (one died and the disease progressed in the other). The transplant-related mortality (TRM) was 22.1% at 2 years and 30% at 5 years, while death during to progression of disease was 3.2% at 2 years and 8.7% at 5 years. Of the 29 surviving patients, 55% achieved complete clinical and laboratory remission and 24% partial remission. The consensus is that nonmyeloablative reduced intensity conditioning regimens should be utilized [37], as will be further discussed dealing with auto-HSCT.

A safe and effective conditioning protocol has been developed in Israel [38], but no lupus patients were transplanted. A large number of SLE patients were allotransplanted in Ahmedabad according to a complicated conditioning protocol [39], but they all relapsed after a mean of 7.35 months of disease-free interval.

A series of mechanisms were considered for the effects of allo-HSCT in ADs, including immunomodulation, tolerization by T regulatory cells and, most importantly immune-mediated destruction of autoreactive cells [40]. By analogy with well-known Graft-versus-Leukemia (GVL) effect [41], this last was defined as a Graft-versus-Autoimmunity (GVA) effect [42]. It was originally found to be more evident when associated with Graft-versus-Host disease (GVHD) [43], but it was not found in the review by Daikeler et al. [36]. Contrarywise, evidence for a GVA effect was demonstrated in models of experimental encephalomyelitis [44]. Mixed chimerism has been thought to be capable of controlling ADs, both in experimental and clinical studies, [45, 46] but in other cases it was accompanied by relapse. The concept that complete remission of ADs depends upon full donor chimerism has been supported by the favorable effect of donor lymphocyte infusions (DLI) for posttransplant relapses, designed to obtain full chimerism. 

Single case reports of SLE and RA patients having undergone allo-HSCT for coincidental diseases are often contradictory. Along with a 20-year complete remission in 2 patients with RA [47] and in 1 with SLE [48], there are also patients with RA who relapsed notwithstanding allo-SCT [49–51].

Donor lymphocyte infusions have been efficacious in controlling incipient relapse [51, 52], but the most disquieting reports are those of patients with SADs having relapsed notwithstanding full donor chimerism [53]. A recent case report concerns a female patient with severe Sjogren's syndrome with associated lupus features [54] complicated by chronic inflammatory demyelinating polyradiculoneuropathy (CIDP) and total inability to walk, who was treated with success for the neurological complication with an auto-HSCT, subsequently developed severe aplastic anemia (SAA), was successfully transplanted from her HLA-identical sister and achieved cure of SAA, but still maintains positive ANA of the speckled type after 5 years [54]. 

The causes of this almost paradoxical behavior are unclear. The persistence of autoantibodies after Auto-HSCT is not an infrequent phenomenon, as will be discussed in the following section. However the relapse of AD notwithstanding the acquisition of a new, healthy immune system is much more intriguing. The persistence of long-lived plasmacells in marrow survival niches [55, 56] has been considered as a possible mechanism of relapse, but their pathogenesis may be even more complex, and the relentless stimulation by self-antigens in genetically autoimmune prone subjects must also be considered [57]. More specifically in SLE, the importance of nucleosome challenge is well ascertained [58].

Still another complication is the occurrence of secondary ADs following HSCT, both autologous and allogeneic [59–61]. Given the absolute preponderance of the autologous versus the allogeneic procedure, it is obvious that most cases have been found in the first category. In the EBMT study 3 patients developed 4 secondary ADs after allo-HSCT, and 13 did not [61]. This number is too small to state that SLE is the disease most liable to develop ADs following allo-HSCT, but this has been confirmed in the autologous setting. Multiple sequential pathogenetic mechanisms have been proposed, but the common features of genetic factors and immune dysregulation are most probably at the origin of this complication. Finally it has been reported that in 5 cases of lupus patients having developed malignant B lymphomas, high-dose chemotherapy (HDCT) was able to eradicate the malignancies, but not SLE [62].

Concluding this section on allo-HSCT for SADs, it must be considered that new clinical studies are under way, in order to explore its efficacy and tolerance. However there can be no doubt that only obtaining a cure can justify its performance. As recently stated by Tyndall [63], “the jury is still out” for a definite judgment. The philosophy of our Center is to offer allo-HSCT to patients with ADs having developed complications such as oncohematological diseases; SAA and others, all requiring the allogeneic procedure.

## 4. Autologous Transplantation

The first two reports of patients with severe, refractory SLE having undergone auto-HSCT were published in 1997 [64, 65]. The patient transplanted in Genoa had a long history of lupus with many severe complications, and has been followed to the time of this writing, making it the longest followup of a single patient (16 years; see Table 1). There followed a series of single case reports, all of them characterized by an extremely severe condition associated with complete refractoriness to conventional therapy. They included patients with refractory SLE in general [66], with severe pulmonary involvement [67], and with complicating Evans' syndrome [68]. Of special interest are two cases of neuropsychiatric SLE (NP-SLE) that were salvaged by auto-HSCT [69, 70]. These case reports paved the way to single center retrospective clinical studies, and subsequently to more extended cooperative ones. They are not to be disregarded, since patients in desperate conditions were rescued by means of the bold and knowledgeable utilization of a procedure until then mostly ignored in this specific area. Single-case reports are known to be classified at the lowest degree of strength in observational studies, but they are considered of interest when reporting “newly recognized or uncommon observations” [71], and this is the case of these pioneering interventions. More extensive clinical trials by dedicated teams were to follow worldwide. They have been resumed in 2 tables, one in the recent summation by Illei et al. [13] and another published in a former contribution by us [72]. Two fundamental findings emerged from these clinical observations, namely the powerful therapeutic effect reported by all centers, and the greatly inferior transplant-related mortality (TRM) as compared to the allogeneic procedure. As discussed with greater detail elsewhere [6], there are three basic questions to be addressed.

### 4.1. Mobilization and Conditioning: Which Are the Best Procedures? 

Hematopoietic stem and early progenitor cells, initially obtained from the bone marrow, and now almost universally from peripheral blood (“mobilized HSC”), are utilized for this procedure. At first there was the suspicion that, in patients with SADs, and even more specifically in SLE, abnormalities of the hematopoietic system, primary or secondary to prolonged immunosuppressive therapy, might have affected their engraftment potential. Accelerated telomeric loss and functional exhaustion have been found in the HSC of rheumatoid arthritis (RA) and of SLE [73, 74], However, recent research in another AD, multiple sclerosis (MS), has shown normal HSC reserves in the bone marrow, largely capable to support hematopoiesis in the autologous transplant setting [75], and this notion has been extended to the majority of SADs, in which the collection of SC is routinary, and their hematopoietic capability is apparently normal, as evaluated by hematological reconstitution.

The main reason for the shift in the collection of HSC from marrow to blood is the larger number that can be harvested, resulting in a faster and stable engraftment [76–78]. T-cell depletion may be performed by *ex vivo* manipulations, but is performed infrequently and only in special cases [79], Cyclophosphamide (CY), with subsequent utilization of granulocyte colony stimulating factors (G-CSF), is the most used drug for mobilization, at the dosage of 2–4 g/m^2^ [79]. Its utilization often allows to achieve a partial remission [78], which in most cases is a favorable prognostic indicator [6]. This observation is in line with the well-known strategy of high dose CY alone performed at John Hopkins University [80, 81], USA.

Conditioning is the conventional term used to indicate the immunosuppressive treatment (combinations of chemo-and radiotherapy) utilized both in allo- and auto-HSCT [82]. While in oncohematological disease there is the double target of reducing to a minimum residue the malignant cells later to be eradicated by the graft's immune activity [40], and to abrogate allogeneic reactivity, in the autologous setting the purpose is the elimination of the autoreactive lymphoid system thought to be at the origin of the AD. This effect practically coincides with the purpose of resetting the immune system, as will be discussed later. When evaluating the most appropriate conditioning regimen for SLE and most other SADs, there are two main aspects to be examined. The first is the clear demonstration that the intensity of the conditioning regimen is usually proportional to its toxicity, but can be inversely proportional to the incidence of relapses. In a retrospective analysis of 450 patients having undergone auto-HSCT for SADs, the different conditioning regimens were divided in high, intermediate, and low intensity, and a significant association was found with intensity and TRM, while an inverse relationship was shown with the incidence of relapse [83]. The second consists in the strategy of utilizing lymphoablative regimens specifically targeting the self-reactive immune system [84, 85]. 

There should not be a real competition between immunosuppressive monoclonals and transplantation in this area. A combination of both strategies, in which 500 mg of the anti-CD20 cell monoclonal Rituximab are administered before and after the usual 200 mg/kg of CY (“sandwich technique”) is currently being utilized for SLE with impressive results, at Northwestern University, Chicago, USA [86]. Anti-CD20 immunotherapy for the control of relapse following auto-HSCT in rheumatoid arthritis has been utilized with success [87], and the strategy of using an additional agent to the transplantation procedure is attractive. However, a devastating complication, progressive multifocal leukoencephalopathy (PML), caused by the activation of the John Cunningham virus, has been reported in a disquieting proportion of patients having been immunosuppressed with biological agents. The first cases were reported in SLE [88], and a recent review reported 52 patients having developed PML, 7 of which following auto-HSCT (3 allogeneic and 4 autologous) for lymphoproliferative disease [89]. This demonstrates that, once again, maximal immunosuppression may lead to unforeseen severe infectious complications.

### 4.2. Is the Procedure Safe, and What Benefits Does It Confer? 

At the time of this writing there are little more than 300 patients having undergone auto-HSCT worldwide. Two tables specifying Centers, results and TRM have been published [12, 13]. TRM varied considerably from center to center. A center effect, similar to the one demonstrated in leukemias, could not be clearly confirmed, but there is evidence of a learning curve. This favorable trend is confirmed in the much greater clinical material composed by SADs in general, in which TRM reached 12% in the first EBMT Registry [83], decreased to 7^+/−^ 3% in 2005, and attained 4% in the Northwestern University's study [86] in SLE. Guidelines and recommendations have been published in detail regarding the choice of the conditioning regimen and the selection of patients [9]. It is obvious that patients with very severe organ damage make poor candidates, and that a patient with end-stage lupus nephropathy is in need more of a kidney than a HSC transplant. However two patients who were already in dialytic treatment recovered renal function following auto-HSCT sufficient to forego dialysis [84]. Although the selection of patients within approved and/or investigational protocols is the best policy, it must be realized that, in selected patients with advanced refractory SLE, the decision to perform auto-HSCT will ultimately rely on a combination of clinical acumen, experienced teams and good patient-doctor relationship.

Coming to the question of what type of benefit does auto-HSCT confer to severe, progressive, refractory-relapsing SLE, more often than not it may be dramatic. In a recent, provocative editorial commenting its utilization in SADs, and more specifically in the rheumatic diseases, Illei has posed the question, whether “the glass is half full or half empty” [90]. We have already given a tentative answer to this question [91], but I shall try to be more specific here.

The idea of obtaining stable complete remissions, if by this term, in analogy with oncohematological diseases, we intend clinical remission, abrogation of all autoimmune markers, and definitive freedom from drug therapy, is not realistic [6, 13, 31, 85, 86]. Independently from the heterogeneity of the clinical material, progression-free survival (PFS), which may be considered as the most accurate estimated outcome of a therapeutic procedure, was 43% at three years in the EBMT study [7]. However very good remissions occur, transplantation may be a salvage treatment in many cases, and in most relapses, often of a milder form than the original disease, the utilization of conventional therapies, to which the patients were formerly refractory, is generally possible. The effects of auto-HSCT may be divided in two phases: the early suppression of ongoing immune-inflammatory events, and the later resetting of the autoimmune clock, which is closely related to the length and grade of remission. The first effect is clearly due to the immunosuppressive conditioning regimens, and is proportional to the dose intensity [83]. No complicated immune dynamics occur here, besides the well-known combination of immunosuppression and abrogation of the attending inflammation. This effect is responsible for the dramatic disease-arresting (“nosostatic”) effects which have been observed in practically all actively aggressive SADs, and most demonstratively in SLE [84, 92]. In the aggressive refractory phases of disease, Auto-HSCT may well be the most potent salvage therapy available. A clear distinction of the diverse sensitivity to auto-HSCT according to the phases of disease has been recently made in multiple sclerosis (MS) by Schevchenko et al. [93], who have divided the transplant phases in MS in “early,” “conventional,” and “salvage-late” procedures. Among the many examples of this early, dramatic therapeutic effect there are, besides the cancellation of systemic symptoms, the almost immediate clearance of inflammatory urinary sediments in lupus nephritis [94], the rapid improvement of nailfold capillaroscopy in SSc [95], and the early abrogation of Gadolinium-enhancing lesions in MS [96]. Intermediate changes may be considered the striking disappearance of diffuse calcinosis in a child with overlap connective disease [97], and the early regression of dermal fibrosis in patients with severe scleroderma [98].

### 4.3. What Significant Changes in the Immune System Take Place following HSCT? Are We Really Curing Autoimmunity?

No other aspect of the Auto-HSCT-based procedures has been the object of so much research, enthusiasm and controversy. A prolonged depression of CD4+ CD45RA cells is a general finding [99], and takes place following both auto-HSCT and high-dose immunosuppressive therapy (HDIS) alone [80, 81]. What type of immunomodulation then follows had been called a “black box” by Muraro and Douek [99], but thanks to their own [100] and others' investigations [101, 102] is becoming increasingly clear. High-dose immunosuppression (HDI) reduces the population of autoimmune cells to a condition which may be considered as minimal residual autoimmune disease (MRAD). While the cure of oncohematological disease requires the eradication of cancer SC, a different view may be entertained for ADs. Two types of immune resetting are now considered, and have been divided in Type I and Type II, according to the modulations of the T/B repertoire and off immune regulation [100].

The first has been defined as a “reeducation” [103] of the faulty immune system, obtained by restoring a diverse antigen-specific repertoire through reactivation of the thymic output (“thymic rebound”), which has been shown to persist, albeit in lesser measure, also in adults. In an immunological study of auto-HSCT in 7 SLE patients the Berlin group has found evidence for an overwhelming regeneration of the B cell lineage, that apparently become tolerant to self-antigens [104]. The recurrence of lupus activity observed in three of these patients was accompanied by the development of antinuclear antibodies with new specificities, a finding they considered as *de novo* development of SLE [105]. Be that as it may, the development of secondary ADs following auto-HSCT has been found to be maximal precisely in SLE [81]. The switch from one to another abnormal balance has been described by Shoenfeld as the kaleidoscope of the autoimmune mosaic [106]. The Type II modality has received a powerful impulse by the recent demonstration that, in 15 post-transplant lupus patients, both CD4^+^ CD25 FoxP3^+^ and an unusual CD8^+^ Fox3^+^ Treg subset return to levels seen in normal subjects [20], accompanied by almost complete inhibition of pathogenic T cell response to critical peptide autoepitopes from histones and nucleosomes. This was not observed in patients in drug mediated remissions, in which CD4T cell autoreactivity to nucleosomal epitopes persisted. Former investigations have also highlighted the role of Tregs in restoring tolerance following auto-HSCT [107].

There are also, however, some controversial results, mostly in other ADs, reporting that autoreactivity did return. In a study of autotransplanted MS patients the T cells recognizing myelin basic protein were indeed initially depleted by immunoablation, but then rapidly expanded from the reconstituted T-cell repertoire in 12 months [108]. An early recovery of CD4^+^T cell receptor diversity was found after Auto-HSCT [109]. In a comprehensive study analyzing original and pooled data from autotransplanted MS patients Mondria et al. [110] found not only the persistence of CSF oligoclonal bands in 88% of the reported cases, but also the persistence of the soluble lymphocyte activator CD27, concluding that complete eradication of activated lymphocytes from the CNS had not been established notwithstanding auto-HSCT and radiation.

Finally, although all these therapies are addressed to eradicate, or just to control, an aberrant, autodestructive immune system, little has been done on the side of the antigens. Available data suggest that the autoimmune response is antigen driven [111], and the consequences of the neo-antigenicity of the “altered self” [112] in genetically disease-prone individuals [113] must be taken into account, especially in patients relapsing after allo-HSCT. A treatment founded on gene therapy-assisted autologous HSC transplantation, with the object of achieving antigen-specific tolerance, is being actively pursued by Alderuccio et al. [114].

## 5. Conclusions and Perspectives

Allogeneic HSCT seemed, at the start of the transplantation saga for SLE, to possess the ability of delivering a 1-hit cure for SLE. Unfortunately this has not been so, and, unless ongoing and future clinical investigations will bring about overwhelmingly solid data, it should be reserved, as in our institution, to patients with so called double trouble [115], that is lupus patients having developed malignant lymphomas and/or other transplant-requiring diseases.

Autologous HSCT has become a promising treatment for severe SLE, and for SADs in general, worldwide. It may be a salvage therapy as well as a disease-controlling procedure. Its effects are both immediate and gradually progressive (“reeducation”). It may turn out to be a robust bridge for more and better biological therapies in the future, similarly to discovery of the tyrosine-kinase inhibitors that have cancelled most allogeneic transplants for chronic myelogenous leukemia (CML).

## Figures and Tables

**Table 1:** A synthesis of the first case of SE performed in Genoa, with a followup of 16 years.

Year	Age	Clinical symptoms	Laboratory tests	Therapy
1983	33	Arthralgias, fever	ANA+ Wasserman test+	NSAIDs
1985	35	Exudative pleuritis pericarditis		Prednisone bolus plus tapered doses
1995	36	Nephropathy proteinuria >10 g/day	ANA 1: 160, ds-DNA pos, LE phenomenon pos, CH50 620, proteinuria, hematuria	CY, prednisone bolus, AZA, auto-HSCT
2000	50	Asthenia, proteinuria 2 g/24 h	ANA 1: 320 homogeneous, ds-DNA neg, complement normal, proteinuria, hematuria	Mycophenolate mofetil 2 g/day, prednisone 1 mg/kg
2005	55	Tendinitis	ANA 1: 320 homogeneous, ds-DNA neg, LE phenomenon neg, complement normal, proteinuria 0.5 g/24 h	Mycophenolate mofetil 2 g/day, prednisone 2 mg/kg plus tapered doses
2008	58	Facial erythema	ANA 1: 320 homogeneous, ds-DNA neg, LE phenomenon neg, complement normal, proteinuria 0.5 g/24 h	Mycophenolate mofetil 2 g/day, prednisone 0.5 mg/kg, hydroxichloroquine
2012	62	Disease quiescent, the patient is well	ANA 1: 680 homogeneous, proteinuria 0.18 g/24 h, complement normal, ds-DNA neg	Mycophenolate mofetil 1 g/day, prednisone 0.5 mg/kg/every other day
